# Natural compounds and mesenchymal stem cells: implications for inflammatory-impaired tissue regeneration

**DOI:** 10.1186/s13287-024-03641-3

**Published:** 2024-02-07

**Authors:** Wen Li, Zichao Xiang, Wenjing Yu, Xiaobin Huang, Qian Jiang, Arwa Abumansour, Ying Yang, Chider Chen

**Affiliations:** 1 Stomatology Hospital, School of Stomatology, Zhejiang University School of Medicine, Zhejiang Provincial Clinical Research Center for Oral Diseases, Key Laboratory of Oral Biomedical Research of Zhejiang Province, Cancer Center of Zhejiang University, Engineering Research Center of Oral Biomaterials and Devices of Zhejiang Province, Hangzhou, China; 2 Department of Oral and Maxillofacial Surgery and Pharmacology, School of Dental Medicine, University of Pennsylvania, 240 S. 40th St., Philadelphia, PA 19104 USA; 3 Department of Orthodontics, School of Dental Medicine, University of Pennsylvania, Philadelphia, PA USA; 4 Department of Endodontics, School of Dental Medicine, University of Pennsylvania, Philadelphia, PA USA; 5 Research and Innovation Oral Care, Colgate-Palmolive Company, Piscataway, NJ USA; 6 Center of Innovation and Precision Dentistry, School of Dental Medicine, School of Engineering and Applied Sciences, University of Pennsylvania, Philadelphia, PA 19104 USA

**Keywords:** Mesenchymal stromal progenitor cells (MSCs), Nature compound (NC), Immunomodulation, Tissue regeneration, Inflammation

## Abstract

Inflammation is a common and important pathological process occurring in any part of the body and relating to a variety of diseases. Effective tissue repair is critical for the survival of impaired organisms. Considering the side effects of the currently used anti-inflammatory medications, new therapeutic agents are urgently needed for the improvement of regenerative capacities of inflammatory-impaired tissues. Mesenchymal stromal stem/progenitor cells (MSCs) are characterized by the capabilities of self-renewal and multipotent differentiation and exhibit immunomodulatory capacity. Due to the ability to modulate inflammatory phenotypes and immune responses, MSCs have been considered as a potential alternative therapy for autoimmune and inflammatory diseases. Natural compounds (NCs) are complex small multiple-target molecules mostly derived from plants and microorganisms, exhibiting therapeutic effects in many disorders, such as osteoporosis, diabetes, cancer, and inflammatory/autoimmune diseases. Recently, increasing studies focused on the prominent effects of NCs on MSCs, including the regulation of cell survival and inflammatory response, as well as osteogenic/adipogenic differentiation capacities, which indicate the roles of NCs on MSC-based cytotherapy in several inflammatory diseases. Their therapeutic effects and fewer side effects in numerous physiological processes, compared to chemosynthetic drugs, made them to be a new therapeutic avenue combined with MSCs for impaired tissue regeneration. Here we summarize the current understanding of the influence of NCs on MSCs and related downstream signaling pathways, specifically in pathological inflammatory conditions. In addition, the emerging concepts through the combination of NCs and MSCs to expand the therapeutic perspectives are highlighted. A promising MSC source from oral/dental tissues is also discussed, with a remarkable potential for MSC-based therapy in future clinical applications.

## Introduction

Inflammation is a physiological state where immune cells evoke a response against detrimental insults, which occurred in any part of the body and relating to a variety of diseases [1]. The inflammation diseases included immune response damages caused by inflammation and autoimmune diseases, such as osteoarthritis, periodontitis, rheumatoid arthritis, and so on [2–4]. Effective tissue repair is critical for the survival of all living organisms [1]. The protection of cell viability and function and the regulation of inflammatory responses are critical for the regeneration of impaired tissues. Some anti-inflammatory drugs, such as aspirin, are able to affect cell function and emerge the tissue remodeling potential [5]; however, the side effects, such as gastric damage, limit their clinical applications [6]. Therefore, new therapeutic agents are urgently needed for the improvement of regenerative capacities of inflammatory-impaired tissues. Autotherapies are a novel treatment strategy to elevate tissue healing and regeneration by inducing the body’s innate ability of itself [7]. The mechanism may be that the host local microenvironment as a stem cell niche provides a unique tissue structure to achieve the endogenous tissue self-healing by activating somatic stem cells [8, 9].

Mesenchymal stromal progenitor cells (MSCs), which express CD44, CD73, CD90, CD105, and SCA-1, but not hematopoietic makers CD45 and CD14, can be isolated from a wide range of tissue sources, including bone marrow, umbilical cord, adipose, liver, as well as orofacial tissues [10]. As one of the adult stem/progenitor cells, MSCs are characterized by the capabilities of self-renewal, multipotent differentiation, and immunomodulation, by which they can regulate the phenotypes, functions, survival, and migration of several types of immune cells, by either cell–cell direct interaction or paracrine pathways such as cytokines and extracellular vesicles [11, 12]. Due to the ability to modulate inflammatory phenotypes and immune responses, MSCs have been considered a potential alternative therapy for autoimmune and inflammatory diseases [12]. In this regard, transplantation of MSCs has been demonstrated in several studies to promote the repairment and regeneration of injured tissues [13–15].

Natural compounds (NCs) are complex small multiple-target molecules found mainly in plants and microorganisms, which have been verified to have therapeutic effects in many disorders, such as osteoporosis, diabetes, cancer, and inflammatory/autoimmune diseases [16, 17]. Some well-known ingredients, such as epigallocatechin-3-gallate (EGCG), resveratrol, and ginsenoside, have been widely studied as effective NC components. These findings identified the highly diverse and specific biological activities of NCs and demonstrated fewer side- or adverse effects in numerous physiological processes compared to chemosynthetic drugs [18–20]. Evidence also revealed the multiple influences of NCs on MSC survival, proliferation, and functions. It was found that resveratrol enhanced the therapeutic effect of PDLSCs by reducing the death of PDLSCs mediated by activated T cells, and thus reduced the dosage of PDLSCs used in transplantation to recover epithelial structure and eliminate inflammatory cells in colitis mice [7]. It was also revealed that Osthole pre-treatment enhanced the BMSC cytotherapy effects in experimental inflammatory colitis and osteoporotic mice [21]. These findings implied the therapeutic effect of treatment combined NCs and MSCs could be better than the treatments involving MSCs alone. Therefore, NCs have been explored for their potential therapeutic effects as complementary/alternative medicines and as functional regulators of MSCs for immunotherapy over the years [22].

Here, we review the current understanding of the roles of NCs in the protection, differentiation, and anti-inflammatory capacities of MSCs, specifically, in the pathological inflammatory conditions, and highlight the emerging concepts through the combination of NCs and MSCs to expand the therapeutic perspectives. (Table 1).

**Table 1:** The components of NCs involved in the review

Natural compounds	Natural plant
*Herbal extracts*	
Astragaloside Calycosin-7-O-β-Glucoside	Extracted from Astragalus, also known as Huangqi in China. It is the dried root of Astragalus mongholicus Bunge. It is widely used as an immune stimulant, an antioxidant, a hepatoprotectant, a diuretic, an antidiabetic, an anticancer drug, and an expectorant. There are more than 100 compounds that have been isolated and identified from this herbal plant.
Salvianolic acids (B&C)	Extracted from the roots of Salvia miltiorrhiza Bunge, also known as Danshen, which is a traditional Chinese medicine for treatment of inflammatory diseases. Salvianolic acid is a polyphenol compound isolated from Danshen and has anti-inflammatory and antioxidant bioactivities.
Ginsenoside *Protopanaxadiol type: Rb1* *Protopanaxatriol type: Rg1*	Extracted from Panax ginseng (PG), which usually refers to the dried root and rhizome of PG CA Meyer of the family Araliaceae. Recently, clinical studies have shown that compounds or medicines containing different forms of PG have a promising effect on side effects caused by chemotherapy. Ginsenosides, which are the main active ingredient of PG, have a pivotal role in the pharmacological actions of PG.
Wedelolactone	Eextracted from Ecliptae herba with the verified therapeutic effects in some bone diseases such as osteoporosis.
Osthole	7-methoxy-8-(3-methyl-2-butenyl) coumarin, which is a bioactive derivative from coumarin.Extracted from Fructus Cnidii, Radix Angelicae Pubescentis, and other traditional Chinese medicines with a wide range of pharmacotherapeutic effects, including the effects of anti-inflammation and positive effects on osteogenic and neuronal differentiation.
*Woody plant-derived bioactive compounds*	
Berberine	A type of quaternary ammonium alkaloid, extracted from varieties of plants species, such as Coptis and Phellodendron. Berberine hydrochloride is typically used in a clinical setting, due to its numerous pharmacological activities, including anti-microbial, glucose/cholesterol regulatory, survival protection, and immune modulatory properties. Berberine also related to bone remodeling, for instance, inhibited adipogenesis and promoted osteogenesis.
Green tea polyphenols: *Catechins* *(EC, EGC, EGCG)* *Gallic acid* Caffeine	Extracted from green tea, a common drink brewed from the dried leaves of Camellia sinensis. The main components are phenolic acids, polyphenols (include catechins and gallic acid), caffeine, minerals, and trace amounts of vitamins, amino acids, and carbohydrates. Catechins accounts for more than 80% of green tea polyphenols and are derived from flavan-3- ol. (−)-epicatechin (EC), (−)-epicatechin gallate (EGC), (−)-epigallocatechin (EGC), and (−)-epigallocatechin gallate (EGCG) are the main types of catechins, possessing the most potent antioxidant and free radical scavenging abilities. Gallic acid is also a natural antioxidant which scavenges the superoxide and hydroxyl radicals and prevents oxidative stress. Caffeine, a key xanthine alkaloid element in green tea, is a type of central nervous system stimulant. The excessive caffeine is a risk factor for osteoporosis and bone fracture.
*Phytoestrogens*	
Resveratrol	A nonflavonoid polyphenol phytoalexin with a stilbene structure, and can be found in multiple plants, including the root of white hellebore (Veratum grandiflorum), polygonum cupsidatum, peanuts, eucalyptus, blueberries, cranberries, and grapes. RSV is an effective antioxidant and closely related to SIRT1 pathway, participating in the modulation of apoptosis, DNA repair, oxidative stress resistance, anti-aging processes, and lipid metabolism.
Naringin	A flavonoid compound commonly extracted from citrus fruits and a traditional Chinese medicine Rhizoma Drynariae, which is usually used to treat osteoporosis and bone fracture.

## The protective effects of NCs on MSCs in inflammatory environment

Various studies have shown that NCs can inhibit the expression of inflammatory cytokines, suppress reactive oxygen species (ROS) accumulation, decrease apoptosis and senescence, and maintain cellular homeostasis in MSCs under an inflammatory environment [23–29]. NCs have protective effects that can create an environment to enhance the survival of MSCs and improve regenerative outcomes, where the biological pro-survival mechanisms play a critical role. (Tables 2, 3).

**Table 2:** The pro-survival effect of NC on MSCs in an inflammatory environment

Natural Compounds	Inflammatory environment/disease	MSC source	Result	Related mechanism/signaling pathway	References
*Astragaloside*					
Astragaloside IV 20–200 µM 40 mg/kg	Iron loading	Bone marrow	Reversed cell viability and proliferation	Iron homeostasis and metabolism	[46]
Astragaloside and Baicalein 100 ng/ml	LPS	Bone marrow	Decreased cell apoptosis	Anti-inflammation (MAPK/ERK↑)	[34]
*Salvianolic acid*					
Salvianolic acid B 10 µM	Hydroperoxide	Bone marrow	Reduced ROS accumulation, attenuated caspase-3 activation, upregulated Bcl-2, and decreased cell apoptosis	MAPK/ERK ↓	[35]
Salvianolic acid C 5 mM	LPS/periodontitis	Periodontal ligament	Reduced oxidative stress markers (ROS, NO, and iNOS) Reversed abnormal apoptotic proteins (BAX, BCL-2, Caspase-3)	TLR4/NF-kB ↓	[33]
*Ginsenoside*					
Ginsenoside Rb1 10^−6^ M	Hydroperoxide	Bone marrow	Showed a good anti-oxidative effect and improved cell viability	N/A	[23]
Ginsenoside Rg1 100 μg/ml	Hypoxia and serum deprivation	Bone marrow	Reversed the upregulated expression of RhoA, ROCK-1, BAD, BAX, and increased the expression of Bcl-2, miR-494-3p	mir-494-3p↑/ROCK-1↓/Bcl-2 ↑	[45]
Ginsenoside Rg1 20 mg/kg	D-Galactose-induced aged rat	Bone marrow	Reversed cell proliferation, and exerted antioxidant effects by reducing the level of ROS and increasing the level of SOD	p16 p53 p21↓	[47]
*Berberine*					
4 μM	Hypoxia and serum deprivation	Adipose tissue	Reduced ROS and reversed cell survival rate Alleviated apoptosis and autophagy	AMPK↓/mTOR autophagy↓	[42]
*EGCG*					
EGCG	Diabetes with pancreatic damage	Adipose tissue	Increased cell viability and survival protein expression, suppressed apoptotic protein markers. Enhanced the therapeutic effect of ADSC injection on pancreatic function	p-AKT↑ SIRT↑( in vivo)	[37]
EGCG 10 μM	Thermal injury	Wharton's jelly	Reduced the number of apoptotic cells, upregulated gene expression of *Bcl2l1* and downregulated *Bax*	ERK↑ AKT↑	[38]
EGCG 5&10&20 μM	Hypoxia	Bone marrow	Inhibited cleaved caspase-3 and caspase-9 and ameliorated hypoxia-caused MSC viability reduction	miR-210 ↑	[62]
*Resveratrol*					
5 μM	LPS	Periodontal ligament	Inhibited PDLSCs apoptosis directly and through the PDLSCs-mediated apoptosis of activated T-cell	ERK/FASL↑ T-cell apoptosis	[7]
15 μg/cm^2^	Sterile inflammation with titanium implants	Bone marrow	Decreased the ROS production	ROS/NF-κB↓	[30]
200 μM	TNF-α osteoporosis	Bone marrow	Rescued the impaired capacity of proliferation and migration	N/A	[29]
25 μM	Osteolysis induced by titanium	Bone marrow	Decreased the caspase-3 levels and reduced cell apoptosis	N/A	[54]
0.5 μM	Pulp-capping material	Bone marrow	Increased cell viability and decreased the percentages of early apoptotic and late apoptotic/necrotic cells	N/A	[25]
0.05 μM	Metabolic syndrome	Adipose tissue	Ameliorated apoptosis, senescence and endoplasmic reticulum stress, and restored proper functions of impaired cells through MVs derived from RSV & azacytydine treated ADSC. Apoptotic genes: *p53, caspase-3, caspase-9, Bax*↓ *Bcl-2*↑ ERs related genes: *Atf-6, Ire-1, Eif2, Perk*↓	N/A	[24]
0.05/5 μM	Metabolic syndrome	Adipose tissue	Increased proliferation, decreased apoptosis and senescence, and suppressed ROS accumulation in ADSC treated with azacytydine together	N/A	[28]

ADSC, adipose-derived mesenchymal stem cells; PDLSC, periodontal ligament-derived stem cells; MSC, mesenchymal stem cells; EGCG, epigallocatechin-3-gallate; LPS, lipopolysaccharide; TNF, tumor necrosis factor; RSV, resveratrol; iNOS, inducible nitric oxide synthase; NO, nitric oxide; ROS: reactive oxygen species; SOD: superoxide dismutase; AKT, protein kinase B; BAD, Bcl-2-associated agonist of cell death; BAX, Bcl-2-associated X; ERK, extracellular signal-regulated kinases; FASL, FAS ligand; MAPK, mitogen-activated protein kinase; NFκB, nuclear factor kappaB; RhoA, ras homolog family member A; ROCK-1, Rho-associated coiled-coil containing protein kinase 1; SIRT 1, sirtuin 1; TLR, Toll-like receptor

**Table 3:** The anti-inflammation effect of NCs on MSCs in inflammatory environment

Natural product	Inflammatory environment/disease	MSC source	Result	Signaling pathway	References
*Astragalus*					
Astragaloside and Baicalein 100 ng/ml	LPS	Bone marrow	Reduced IL-1β, IL-8, and TNF-α levels	MAPK/ERK ↑	[34]
*Salvia miltiorrhiza Bunge*					
SAC 5 mM	LPS/periodontitis	Periodontal ligament	Reduced inflammatory cytokines (TNF-α, IL-6, and IL-1β)	TLR4/NF-kB ↓	[33]
*Ginsenoside*					
Ginsenoside Rg1 (Rg1) 20 mg/kg	D-Galactose-induced aged rat	Bone marrow	Reduced the inflammatory cytokine secretion (IL-2, IL-6 and TNF-α) and increased stem cell factor (SCF)	p16 p53 p21↓	[47]
*Osthole*					
10^–5^ mol/L	Osteoporosis	Bone marrow	Restored the immunosuppressive ability to induce T-cell apoptosis and improved the BMSC cytotherapy efficacy in experimental inflammatory colitis and osteoporosis	FasL/Fas ↑ T-cell apoptosis	[21]
*Berberine*					
1–10 μM	LPS	Bone marrow	Reversed the gene expression of pro-inflammatory factors (*Mcp-1, Tnf-α, Il-6, and Il-1β*)	NF-κB ↓	[31]
*EGCG*					
EGCG 10 μM	Triple-negative breast cancer	Adipose tissue	Inhibited expression of inflammatory signaling pathways (*Il-1β, Il-6, Vegf-α, Hif-1α, Cox2*) and suppressed cell migration induced by the cancer cell secretome		[77]
EGCG 10 μM	Thermal injury	Wharton's jelly	Upregulated gene expression of *Tgf-β1*, *Il4* and significantly downregulated expression of *Il-6*, *Il1β*	ERK↑ AKT↑	[38]
*Resveratrol*					
5 μM	Colitis periodontitis	Periodontal ligament	Inhibited inflammatory T-cell infiltration, enhanced the therapeutic effect of PDLSCs transplantation, recovered epithelial structure in colitis mice, and rescued bone loss in periodontitis mice	FasL/Fas↑ T-cell apoptosis	[7]
20–200 μM	TNF-α Osteoporosis	Bone marrow	Decreased TNF-Α-induced inflammatory cytokine expression (*IL-6, MMP-9, IL-1Β*) in a dose-dependent manner	N/A	[29]
0.5 μM	Pulp-capping material	Bone marrow	Regulated inflammatory related genes: *IL-8*↓*, IL-10*↑*, HBD-2*↑*, and BCL-2*↑, and attenuated inflammatory process	N/A	[25]
0.05 μM	Metabolic syndrome	Adipose tissue	Regulated inflammatory secretion: TNFα↓ IL-10↑, and restored proper functions of impaired cells through MVs derived from RSV & azacytydine treated ADSC	N/A	[24]
50 μM	TNF-α induced inflammatory response	Bone marrow	Attenuated TNF-α induced inflammatory response by reducing IL-6, IL-1β, MMP-9, MCP-1	SIRT1↑	[40]
50/100 μM	TNFα-induced inflammatory response	Dental pulp	Suppressed mRNA expression of *Il-6, Il8* Activated autophagy	TNFα/JNK/MAPK ↓ autophagy↑	[41]
0.05 μM	Metabolic syndrome	Adipose tissue	Increased the T_REG_ number, induced mitophagy in PBMCs and decreased levels of TNF-α, NO, and IL-6 in RAW264.7 co-cultured with RSV & azacytydine treated ADSC	N/A	[26]
< 50 µM	IL-1β-induced inflammatory response	Bone marrow	Inhibited *Mmp 13* expression induced by IL-1β	N/A	[27]

ADSC, adipose-derived mesenchymal stem cells; PDLSC, periodontal ligament-derived stem cells; PBMC, peripheral blood mononuclear cells; T_REG_, T lymphocytes; EGCG, Epigallocatechin-3-gallate; LPS, lipopolysaccharide; SAC, salvianolic acid C; RSV, resveratrol; NO, nitric oxide; COX2, cyclooxygenase 2; HIF, hypoxia-inducible factor; IL, interleukin; MCP, monocyte chemotactic protein; MMP, matrix metalloproteinase; TGF, transforming growth factor; TNF, tumor necrosis factor; IL, interleukin; VEGF, vascular endothelial growth factor; AKT, protein kinase B; ERK, extracellular signal-regulated kinase; FASL, FAS ligand; JNK, c-Jun N-terminal kinase; MAPK, mitogen-activated protein kinase; NFκB, nuclear factor kappaB; SIRT 1, sirtuin 1; TLR, Toll-like receptor

### NF-κB pathway

**Fig. 1:**
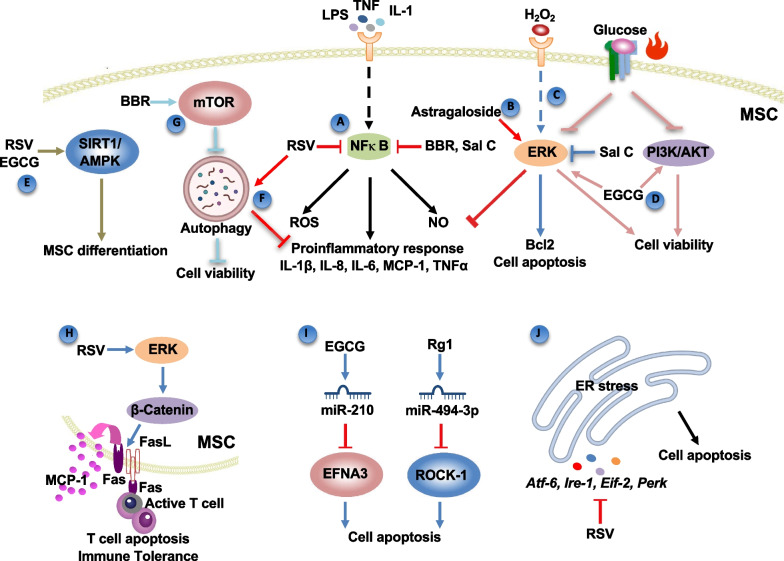
Signaling pathways involve in the viability of MSCs after treated with nature compounds. **A** NC treatment can inhibit pro-inflammatory NFκB pathway. **B** ERK-MAPK pathway is involved in the inhibition of pro-inflammatory cytokines. **C** Sal B prevents cells from apoptosis stimulated by H2O2 through the suppression of the ERK-MAPK pathway. **D** Inhibition of either ERK/MAPK or PI3K/AKT pathway leads to a significant decline in cell viability. **E** Sirt1 pathway significantly mediates the regulation of resveratrol on MSCs differentiation. **F** Resveratrol protects cells through negatively regulating the expression of inflammatory cytokines by autophagy. **G** mTOR signaling regulated by NC treatment is one of the most important metabolic checkpoints controlling cell death and the cross talk between apoptosis and autophagy. **H** Cross talk between ERK and Wnt pathways via resveratrol treatment regulates immunomodulation of MSCs. **I** miRNAs are essential for the protective effects on BMSCs. **J** Resveratrol ameliorates MSC viability through suppression of ER stress. This figure is created with BioRender.com

The nuclear factor kappaB (NF-κB) pathway has a vital role in regulating the expression of genes related to cell survival, differentiation, inflammation, and apoptosis. It can be activated by various stimuli in different inflammatory environments, such as lipopolysaccharides (LPS), tumor necrosis factor (TNF), interleukin-1 (IL-1), and ROS. Studies showed that NF-κB pathway participated in the effects of NCs on oxidative stress and inflammation in MSCs. Resveratrol (RSV) is a nonflavonoid polyphenol phytoalexin extracted from multiple plants, including the root of white hellebore (Veratrum grandiflorum). A coating of RSV-loaded titania nanotube was demonstrated to reduce ROS production and suppress ROS accumulation in bone marrow MSCs (BMSCs), which was related to the phosphorylation inhibition of the downstream NF-κB pathway [30]. The activation of NF-κB signaling and the enhancement of mRNA transcription of pro-inflammatory factors were also effectively ameliorated in the LPS-treated BMSCs by Berberine (BBR), a main extract from Coptis and Phellodendron (Fig. 1A) [31]. The inflammation mediators, including MCP-1, TNF-α, IL-6 and IL-1β, were notably reversed back to the basic level in the BMSCs. Salvianolic acid C (Sal C) is a polyphenol compound isolated from Salvia miltiorrhiza Bunge (Danshen) [32]. It was reported that in the LPS-treated periodontal ligament-derived MSCs (PDLSCs), Sal C reduced the increased levels of oxidative stress markers, including reactive oxygen species (ROS), nitric oxide (NO), and inducible nitric oxide synthase (iNOS), and reverse the abnormal apoptotic proteins such as B cell lymphoma-2 (BCL-2), BCL-2-associated X (BAX), and Caspase-3 [33]. These pro-survival effects were accompanied by the suppressed phosphorylation of NF-kB p65 and the reduced expression levels of Toll-like receptor 4 (TLR4), which were abolished by the overexpression of TLR4 while strengthened by the interference of TLR4 expression and NF-kB inhibitor.

### MAPK/ERK pathway and PI3K/AKT pathway

The mitogen-activated protein kinase (MAPK)/extracellular signal-regulated kinase (ERK) pathway has been reported to be involved in many pharmacological functions, including anti-inflammation and anti-apoptosis. The role of the ERK pathway in protecting MSC by different NCs is not consistent. Astragaloside, a pure compound extracted from astragalus, inhibited the expression of IL-1β, IL-8, and TNF-α in LPS-stimulated BMSCs with the combination of baicalein, while the anti-inflammatory effect was recovered with ERK inhibitor (U0126), which partially indicated MAPK/ERK pathway activation has participated in the astragaloside regulated inflammatory response (Fig. 1B) [34]. However, in the hydroperoxide condition, Sal B reduced ROS accumulation and caspase-3 activation, and inhibited the ERK1/2 phosphorylation in BMSCs, leading to upregulated BCL-2 and decreased cell apoptosis. The effect of Sal B was equivalent to the MAPK/ERK inhibitor (PD98059), indicating that Sal B could prevent cells from apoptosis stimulated by H_2_O_2_ through the suppression of the MAPK/ERK pathway (Fig. 1C) [35].

The phosphoinositide 3-kinase (PI3K)/protein kinase B (PKB/AKT) pathway is considered a signaling cascade independent of the ERK pathway, but the activation of AKT and ERK can often be affected by communal substrates to arouse various cell responses, including cell proliferation, metabolism, apoptosis, development, and differentiation [36]. In the high glucose or the thermal stress, the survival/apoptotic genes were reversed in MSCs derived from adipose tissue and Wharton's jelly treated with Polyphenol (-)-epigallocatechin-3-gallate (EGCG), the most abundant polyphenol in green tea [37]. The increased cell viability and reduced apoptotic rate were observed after EGCG treatment with the prevention of heat-induced decrease of ERK 1/2 and AKT phosphorylations, and the inhibition of either MAPK/ERK or PI3K/AKT led to a significant decline in cell viability (Fig. 1D) [38].

### Sirt1 pathway

The sirtuin1 (Sirt1), a well-known longevity-related protein, is able to epigenetically modulate diverse gene expressions by deacetylation of the nuclear protein histone [39], and can function as an antioxidant through several proteins, including the AMP-activated protein kinase (AMPK) and p-AMPK proteins [37]. As a potent Sirt1 activator, RSV was reported to exert anti-inflammation property via Sirt1 pathway. RSV significantly enhanced the expression level of Sirt1 and inhibited the release of IL-6, IL-1β, MMP-9, and MCP-1 in the culture medium of TNF-α treated BMSCs [40]. In addition, Sirt1 and p-AMPK proteins were found to be elevated in pancreatic tissues in rats with type 1 diabetes after the EGCG-treated adipose tissue-derived MSCs (ADSCs) injection, resulting in significant improvement of damaged pancreatic tissues regeneration [37]. Indeed, Sirt1 pathway also significantly mediated the regulation of RSV on MSCs differentiation (Fig. 1E).

### Autophagy

Autophagy has been reported to control inflammation by inhibiting inflammasomes that promote the maturation of inflammatory mediators and regulating pro-inflammatory cytokines at the transcriptional level, which was also reported to be related to cell apoptosis. RSV was revealed to suppress mRNA expression of* Il-6, Il8* in TNFα-treated dental pulp stem cells (DPSCs), which was demonstrated to be related with autophagy-mediated c-Jun N-terminal kinase (JNK) pathway. The activation of autophagy by RSV was correlated with reduced JNK phosphorylation, while the deactivation of autophagy by using Atg5 siRNA was correlated with enhanced phosphorylation of JNK and augmented increase in *Il-6 and Il8* transcription. These findings indicated that autophagy negatively regulated JNK phosphorylation and the following expression of inflammatory cytokines, while RSV protected cells from TNFα partly by inhibiting JNK pathway through the activation of autophagy in DPSCs (Fig. 1F) [41]. On the contrary, the activation of autophagy was demonstrated to be positively correlated with apoptosis. In the hypoxia and serum deprivation condition, the BBR treatment directly reduced ROS levels, regulated apoptotic proteins, and thus alleviated cell apoptosis in ADSCs, where the increased p-AMPK and decreased p-mTOR induced by starvation were reversed by BBR [42]. This anti-apoptotic effect of BBR was demonstrated to be disturbed by the activator of autophagy (rapamycin), indicating BBR contributed to cell survival by alleviating autophagy through the regulation of AMPK-mTOR pathway, which is one of the most important metabolic checkpoints controlling cell death and the junction of the cross talk between apoptosis and autophagy (Fig. 1G).

### FasL/Fas mediated T-cell immunosuppression

Fas ligand (FasL) is a type II transmembrane protein binding with Fas to form the death-inducing signaling complex. The FasL/Fas pathway belongs to the extrinsic apoptotic pathway, leading to the recruitment and activation of initiator caspases such as caspases 8 and 10 [43]. Recently, FasL/Fas signaling was found to be important for the immunomodulatory ability of MSCs, which is also one of the mechanisms by which NCs regulate the inflammatory response. In the experimental osteoporotic mice, the expression of Fas and FasL in BMSCs was downregulated, resulting in a significant decrease in the ability of BMSCs to induce T-cell apoptosis. Therefore, the treatment by injecting these BMSCs derived from osteoporotic mice was ineffective for experimental inflammatory colitis and osteoporosis. Osthole is a natural pyroxanthin originally extracted from the Cnidium plant, which has been proven to have various functions, such as osteogenesis, anti-inflammation, and so on. The pre-treatment of Osthole rescued the Fas and FasL expression in osteoporotic BMSCs effectively, increased the apoptosis rate of co-cultured T cells, and thus largely enhanced the efficacy of BMSCs cytotherapy [21]. RSV also enhanced the immunomodulatory property of PDLSCs of mediating apoptosis of activated T cells and inhibiting their infiltration through FasL/Fas signaling pathway [26]. In addition, the RSV-treated PDLSCs showed higher resistance to the killing effect of activated T cells. The elevation of FasL by RSV was accompanied by the increase of ERK and active β-catenin, and the knockdown of ERK dramatically reduced the level of FasL, indicating that RSV-regulated immunotherapy in PDLSCs was related to the cross talk among FasL, ERK, and β-catenin signaling (Fig. 1H).

### miRNAs

Numerous studies showed that microRNAs (miRNAs) are involved in cell development and differentiation [44]. miRNAs are also able to influence the expression of key signals in MSCs, such as caspase and Rho kinase, regulating cell apoptosis and release of inflammatory cytokines. Studies found that EGCG could upregulate miR-210 in BMSCs via targeting the 3’UTR of ephrin-A3 (EFNA3), which blocked the hypoxia-induced apoptosis and inhibited the cleaved caspase-3 and caspase-9. Ginsenoside Rg1, a bioactive component of Ginseng and Panax Notoginseng, has demonstrated anti-inflammatory and anti-apoptotic effects. Ginsenoside Rg1 has been shown to have protective effects on BMSCs apoptosis in hypoxia and serum deprivation condition. It inhibited the expression of Rho-associated coiled-coil containing protein kinase 1 (ROCK-1), myosin light chain 2 (MLC-2), Bcl-2-associated agonist of cell death (BAD) and BAX through activating the expression of BCL-2 and miR-494-3p. However, Rg1 no longer altered their expression after ROCK-1 knockout, and loss the function to change ROCK gene expression after the inhibition of miR-494-3p. In addition, the anti-apoptotic effect of Rg1 disappeared after mir-494-3p suppression. These findings indicated mir-494-3p and the related ROCK-1 signaling pathway were essential for the protective effect of Rg1 on BMSCs (Fig. 1I) [45].

### Homeostasis/ER stress/senescence signals

Except the antioxidant effects which has been discussed in the previous content, the regulation of NCs on intracellular homeostasis, senescence, and endoplasmic reticulum (ER) stress also contributes to the mechanism of the protective effects. Astragaloside was demonstrated to diminish cell apoptosis by maintaining ionic homeostasis and metabolism in the iron-loading condition [46]. In the D-Galactose-induced aged rats, BMSCs derived from the Rg1-treated rats showed declined level of ROS and less inflammatory markers, such as IL-2, IL-6, and TNF-α, which was accompanied by the suppression of the senescence-associated proteins including p16, p53, and p21 [47]. In the metabolic syndrome-derived MSCs, the decreased ER stress-related genes, including *Atf-6, Ire-1, Eif2, and Perk*, were observed in cells cultured with the extracellular microvesicles (MV) derived from RSV and azacytydine co-treated ADSCs [24]. Additionally, the expression of apoptotic genes, such as *p53, caspase-3, caspase-9,* and *Bax* were inhibited, while *Bcl-2* was increased, indicating the RSV-ameliorated cell apoptosis partly through the suppression of ER stress (Fig. 1J). Taken together, NCs have been shown as an effective next-generation medication to improve MSC-based tissue regeneration through complex signaling networks to either protect MSC survival or inhibit pro-inflammatory responses (Fig. 1).

## The effects of NCs on the differentiation of MSCs in the inflammatory environment

The effects of NCs on MSC differentiation under the inflammatory process were mostly focused on the capabilities of osteogenesis and chondrogenesis, in which several signaling pathways participate in this process. (Table 4).

**Table 4:** The effect of NCs on MSCs differentiation in inflammatory environment

Natural product	Disease/back ground	MSC source	Effect	Results	Related mechanism /signaling pathway	References
*Astragalus*						
Astragaloside IV 20–200 µM 40 mg/kg	Iron loading	Bone marrow	Osteogenic differentiation↑ Adipogenic differentiation↓	Reduced adipogenesis and increased the osteogenic-related proteins (OCT4, SOX2, OCN, ALP) Reduced bone loss in mice	Iron homeostasis and metabolism	[46]
*Salvia miltiorrhiza Bunge*						
SAC 5 mM	LPS	Periodontal ligament	Osteogenic differentiation	Enhanced ALP activity and the ability of mineralization, osteogenic-related proteins (BMP-2, OCT4, SOX2, RUNX2)	N/A	[33]
*Ginsenoside*						
Rb1 10^−8^ M/10^−6^ M 3 or 6 mg/kg	OVX osteoporosis	Bone marrow	Osteogenic differentiation	Increased ALP activity, mineralization, and the expression of osteogenic-related proteins in normal BMSCs in vitro; but did not have an effect on bone loss, dyslipidemia, and excessive oxidative stress in OVX osteoporosis rats	N/A	[23]
*Wedelolactone*						
2 μg/mL	TNF-α	Dental pulp	Osteogenic differentiation	Promoted the nuclear accumulation of β-catenin, stimulated the expression of odontoblast-related marker genes (*Dmp1, DSPP, Runx2*)	Sema3A/NRP1/β-catenin↑ TNF-α/NF-κB↓	[49]
*Osthole*						
50–100 μM	Alcohol/alcohol-induced ONFH	Bone marrow	Osteogenic differentiation↑ adipogenic differentiation↓	Rescued ALP activity and the expression of osteogenic genes (*Col-I, Ocn and Opn*) and proteins (RUNX2, COL-I, OCN) Decreased the expression of pivotal adipokines (Leptin and PPARγ)	Wnt/β-catenin ↑	[48]
10^–7^ M	Periodontitis	Periodontal ligament	Osteogenic differentiation	Reversed the expression of osteogenic-related genes and proteins (ALP, RUNX2, OSX), increased ALP staining and mineralization	MOZ & MORF ↑ Histone acetylation (H3K9 &H3K14)	[60]
*Berberine*						
1–10 μM	LPS	Bone marrow	Osteogenic differentiation↑ Adipogenic differentiation↓	Reverses the LPS-induced decrease in osteogenic gene expression and increase in adipogenic gene expression levels	AMPK ↑	[31]
*EGCG*						
0.52 μg/kg	Bone fracture	Tibial fracture mice	Osteogenic differentiation	Enhanced callus formation, increased bone volume, and subsequently improved the mechanical properties of the tibial bone	BMP-2 ↑	[63]
5–40 μM	Hypoxia	Bone marrow	Osteogenic differentiation	Upregulated the hypoxia-suppressed expression of RUNX2, BMP-2, ALP, and PINP	miR-210 ↑	[62]
*Resveratrol*						
200 μM	TNF-α	Bone marrow	Osteogenic differentiation↑ Adipogenic differentiation↓	Upregulated ALP and Alizarin Red accumulation and osteoblast-related factors expression (COL-I, RUNX2) Downregulated Oil Red O accumulation and lipid drops	Hippo ↓ YAP/RUNX2 ↑	[29]
25 μM	Osteolysis induced by titanium	Bone marrow	Osteogenic differentiation	Promoted osteogenic differentiation and enhanced bone microstructure around the prosthesis. Enhanced the osteogenic genes (*β-catenin, Runx2, Ocn)*	Wnt/β-catenin ↑	[54]
15 μg/cm^2^	Sterile inflammation with titanium implants	Bone marrow	Osteogenic differentiation	Enhanced the expression of osteogenesis-related genes (*Ocn, Runx2, Opn, and Col-I*), ALP activity and calcium deposition	NF-κB ↓	[30]
5 μM	TNF-β	Adipose tissue	Osteogenic differentiation	Reversed TNF-β-promoted impairments in MSCs osteogenesis	TNF-β/ NF-κB ↓ SIRT 1/Runx2↑	[56]
1 μM	Amyotrophic lateral sclerosis	Bone marrow	Neuronal differentiation	Increased neuroprogenitor markers expression (*nestin, Musashi, CD133, GFAP*) and promoted the neurite numbers and lengths	SIRT 1/AMPK↑	[39]
10 nM	TNF-α Periodontitis	Periodontal ligament	Osteogenic differentiation	Preserved the aggregate formation ability and osteogenesis	NFκB ↓ AMPK ↑	[55]
5 µM	TNF-α	Dental pulp	Osteogenic differentiation	Upregulated the expression of RUNX2, BMP-2, COL-I, and enhanced ALP staining in condition of 10 ng/ml TNF-α	SIRT1-Wnt/β-catenin ↑	[53]
< 50 µM	IL-1β	Bone marrow	Chondrogenic differentiation	Maintained chondrocyte markers (*Col2, aggrecan* and *Sox9*) mRNA expressions in inflammation	N/A	[27]
*Naringin*						
0.1 µM	Hydroperoxide	Adipose tissue	Osteogenic differentiation	Reversed the H_2_O_2_-induced decrease in ALP activity, osteogenic gene expression (*Runx2, Osx*)	Wnt/β-catenin ↑	[52]
0.1 µM	TNF-α	Bone marrow	Osteogenic differentiation	Rescued the TNF-α-induced decrease in ALP activity, osteogenic gene expression (*Runx2, Osx*)	NF-κB↓	[51]

EGCG, epigallocatechin-3-gallate; GRb1, Ginsenoside Rb1; IL, interleukin; LPS, lipopolysaccharide; OVX, ovariectomy; ONFH, osteonecrosis of the femoral head; SAC, salvianolic acid C; TNF-α, tumor necrosis factor α; ALP, alkaline phosphatase; BMP-2, bone morphogenetic protein 2; COL-I, collagen I; DMP1, dentin matrix protein-1; DSPP, dentin sialophosphoprotein; OCN, osteocalcin; OPN, Osteopontin; OSX, osterix; PINP, propeptide of type I procollagen; PPARγ/Pparg, peroxisome proliferator-activated receptor γ; RUNX2, Runt-related transcription factor 2; SOX, sex-determining region Y-box; TRAP, tartrate-resistant acid phosphatase; AMPK, adenosine monophosphate-activated protein kinase; ERK, extracellular signal-regulated kinases; JNK, c-Jun *N*-terminal protein kinase; NF-κB, nuclear factor kappaB; SIRT1, sirtuin 1; Pgc1α, peroxisome proliferator-activated receptor gamma coactivator-1α; YAP, yes-associated protein

### Wnt/β-catenin pathway and NF-κB pathway

**Fig. 2:**
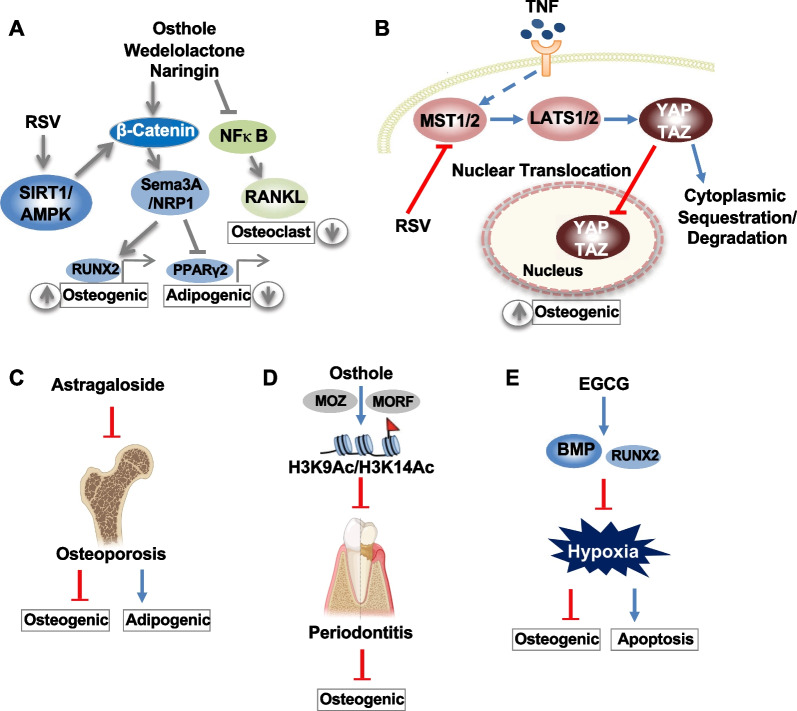
Signaling pathways involve in the differentiation of MSCs after treated with nature compounds. **A** NFκB and Wnt signaling pathways are involved in the differentiation of MSCs. **B** YAP expression and nuclear translocation mediated by resveratrol directly enhances osteogenesis of MSCs. **C** Astragaloside treatment ameliorates osteoporosis through re-balancing the osteogenesis and adipogenesis. **D** Osthole-mediated histone modifications rescue bone loss phenotypes in periodontitis. **E** EGCG treatment ameliorates hypoxia-induced bone loss via activation of osteogenic genes. This figure is created with BioRender.com

Wnt/β-catenin and NF-κB pathways are well known in regulating osteoblastic and osteoclastic differentiation, respectively. It was revealed that the Wnt/β-catenin pathway participates in the rescue of ethanol-induced inhibition on osteogenic differentiation of BMSCs by Osthole. The expression levels of osteocalcin (OCN), type 1 collagen (COL-I), and β-catenin were significantly decreased in BMSCs after ethanol treatment, as well as Alizarin red staining and alkaline phosphatase (ALP) staining. However, Osthole reverses ethanol-induced inhibition of β-catenin levels and osteogenic proteins in a dose-dependent manner. The use of Wnt antagonist (JW74) demonstrated the importance of Wnt/β-catenin cascades for osteoprotective function by Osthole. It has been revealed that the Wnt antagonist dramatically abolished the upregulation of β-catenin and extracellular mineralization when BMSCs were co-treated with ethanol and Osthole [48]. In the TNF-α induced inflammatory condition, the wedelolactone was also able to promote the nuclear accumulation of β-catenin and the expression of odontoblast-related genes (*Dmp1, DSPP* and *Runx2*) in DPSCs, resulting from the activation of semaphorin 3A (Sema3A) and its receptor neuropilin-1 (NRP1) through enhancing canonical Wnt/β-catenin pathway. The effect of wedelolactone was accompanied by the upregulation of the levels of IκBα and the inhibition of the phosphorylation and nuclear migration of p65; thus, the suppressed TNF-α/NF-κB signaling led to the inhibition of receptor activator of nuclear factor-κB ligand (RANKL)-induced osteoclastogenesis and promoted osteoblastogenesis indirectly [49, 50]. Furthermore, Naringin, a flavonoid compound that is commonly found in citrus fruits and a traditional Chinese medicine Rhizoma Drynariae, rescued the decreased osteogenic gene expression (*Runx2, Osx*) and ALP activity induced by the TNFα or hydroperoxide. These effects were accompanied by the suppression of NF-κB and blocked in the presence of Wnt inhibitor DKK-1, suggesting that the effect of naringin on osteogenesis was related to the NF-κB and Wnt signaling pathway (Fig. 2A) [51, 52].

### Sirt1 pathway

Sirt1 pathway is also closely associated with Wnt/β-catenin, NF-κB, AMPK, RUNX2, and PPAR-γ, resulting in the promotion of osteogenic differentiation and the inhibition of adipogenesis. Studies found that the activation of Sirt1 elevated osteogenic markers with the enhancement of the Wnt/β-catenin signaling pathway in MSCs treated with TNF-α or titanium particles [53, 54]. It was also found that RSV, the activator of Sirt1, enhanced the osteogenic genes and decreased the upregulated p-NFκB p65 in MSCs stimulated by TNF α/TNF β or titanium, and thus preserved the aggregate formation ability and reversed the osteogenesis of the MSCs/PDLSCs under inflammatory cytokine treatment [30, 55, 56]. Interestingly, the suppressed p-AMPK levels were found in PDLSCs under inflammatory conditions or in the inflammatory-impaired PDLSCs, while RSV significantly reversed AMPK phosphorylation and osteogenesis (Fig. 2A). In nicotinamide-treated ADSCs, pre-treatment with RSV significantly enhanced osteogenesis by increasing expression of Runx2 and decreasing expression of PPAR-γ, as well as adipogenesis. The activation of Sirt1 in ADSCs increased its binding to PPAR-γ and repressed PPAR-γ activity, which is mediated partly by Sirt1/Runx2 association and deacetylation of Runx2, leading to a dysfunction of the pivotal adipokines. In addition, the Sirt1/AMPK and β-catenin activities could also participate in the regulation of chondrogenic and neuronal differentiation by RSV in MSCs [27, 39].

### Hippo/YAP/Runx2 pathway

The Yes-associated protein (YAP) signals play a critical role in controlling cell lineage commitment and migration capacity of BMSCs. It has been reported that the mammalian sterile 20-like kinase 1/2 (MST1/2) and large tumor suppressor 1/2 (LATS1/2), the core cascade of Hippo kinases, were remarkably upregulated and overactivated in TNF-α condition, leading to an obvious decrease in the nuclear expression of YAP. The over-activating Hippo kinases and the decreasing nuclear YAP in the inflammatory environment could be markedly normalized by RSV treatment, and the YAP-mediated osteogenesis was rescued accordingly. Interestingly, although RSV was able to attenuate inflammation, the inhibitor of YAP obviously decreased the nuclear expression of Runx2, but not affect the inflammatory cytokine expression, which indicated that YAP expression and nuclear translocation mediated by RSV directly enhanced osteogenesis of BMSCs through the regulation of Runx2 without the influence on inflammatory cytokines (Fig. 2B) [29].

### Metabolic reconfiguration

Differentiation is clearly an energy-demanding process, and recent study suggested that extensive metabolic reconfiguration occurred in the MSCs self-renewal and differentiation [57]. Curcumin is a natural lipophilic compound that displays abilities to enhance tissue regeneration and inhibit inflammatory conditions in several tissues [58]. Our previous study found that curcumin significantly promotes PDLSC self-renewal and multipotent differentiation capabilities by activating ERK and mTOR cascades through upregulating growth factor pathways for metabolic reconfiguration toward glycolysis [59]. Meanwhile, PDLSCs immunomodulation is also significantly increased after curcumin treatment through activation of prostaglandin E2-Indoleamine 2,3 dioxygenase signaling, whereas inhibition of glycolysis activity by 2-deoxyglucose largely blocked immunomodulatory capacity of PDLSCs [59].

### Other mechanisms

Homeostasis also contributed to osteogenesis mediated by NCs. In the osteoporosis mice, Astragaloside IV was revealed to inhibit the bone loss derived by iron dextran via the regulation of iron homeostasis and metabolism. The BMSCs isolated from the Astragaloside IV-treated osteoporotic mice have markedly rescued the expression of osteogenic markers and restored the function of osteogenic differentiation [46]. In addition, the adipogenesis of the osteoporotic BMSCs was significantly suppressed (Fig. 2C). The osteogenic regenerative ability of impaired PDLSCs derived from periodontitis tissues could be reversed by Osthole, in which the upregulated histone acetylases MOZ (monocytic leukemia zinc finger protein) and MORF (MOZ-related factor) specifically catalyzing acetylation of Histone3 lisine9 (H3K9) and Histone3 lisine14 (H3K14) were the key regulators in the Osthole rescued osteogenesis (Fig. 2D) [60]. However, in a normal condition without inflammation or other pathological conditions, there are contradictory findings showing the Osthole could exhibit an anti-proliferative and anti-osteogenic role to keep MSCs in a quiescent state. In this regard, Osthole induced an accumulation of cells at G0/G1 phase and a corresponding decrease at the S phase in the cell cycle regulation. This anti-proliferative effect was confirmed by a reduction in the expression of proteins PCNA (proliferating cell nuclear antigen) and CyclinD1 after Osthole treatment [61]. Under the hypoxia condition, miR-210 could be restored by EGCG in MSCs, and the decreased osteogenic proteins, including BMP-2 and Runx2, were also upregulated after EGCG treatment, which ameliorated hypoxia-induced apoptosis [62]. In the tibial fracture mouse model, percutaneous injection of EGCG strongly elevated BMP-2 and enhanced the callus formation, which subsequently improved the bone mechanical properties (Fig. 2E) [63]. In addition, the treatment of EGCG induced ROS production and reduced glutathione levels via 67-kDa laminin receptor (67LR) pathways, which also triggered the apoptosis during adipogenic differentiation, hence leading to the inhibition of adipogenesis in BMSCs [64–66].

## The new insights on MSC-based treatment for inflammatory-impaired tissue regeneration

The cytotherapy of MSC transplantation has been regarded as a safe and effective treatment to improve tissue repair and regeneration. In recent years, numbers of studies showed the effects of NCs on the proliferation, anti-inflammation, and differentiation of MSCs in vivo, and demonstrated that the pre-treatment or co-treatment with NCs exhibited a significant promotion in the therapeutic effects of MSCs (Table 5). Thus, the MSC therapy combined with NCs has been fronted as a promising treatment for inflammatory-impaired tissue regeneration.

**Table 5:** The therapeutic effect of NCs on MSCs transplantation in vivo

Natural product	Inflammatory environment /disease	MSC source	MSCs therapy	Result	Signaling pathway	References
*Astragalus*						
Ethanolic extract of astragalus (EEA)	LPS/ulcer mice	Wharton’s jelly	Sustained-release gel with MSC exosomes + EEA	Reduced the expression of inflammatory factors and increased IL-10 in macrophages in vitro Decreased macrophages aggregation and the content of IL-1β and IL-6 in the peripheral blood in vivo	N/A	[72]
*Ginsenoside*						
Ginsenoside Rg1 10 μM	Radiation induced intestinal injury	Bone marrow	Injection of condition medium of MSCs treated by Rg1	Downregulated inflammatory responses (IL-1β and TNF-α) in jejunal protein of irradiated rats and in irradiated IEC-6 cells in vivo and in vitro	HO-1 ↑	[68]
*Osthole*						
10^–5^ mol/L	Osteoporosis	Bone marrow	Injection of Osthole-treated MSCs	Restored immunosuppressive ability Reduced lymphocyte infiltration and mucosal damage in experimental inflammatory colitis and prevented cortical bone damage and bone microstructure damage in osteoporosis model	Fas/FasL ↑ T-cell apoptosis	[21]
10^−5^ M	OVX osteoporosis	Bone marrow	Injection of Osthole-treated MSCs	Elevated serum levels of P1NP, ALP, and calcium, reduced serum level of TRAP, and enhanced the therapeutic effect of BMSC injection on bone loss in OVX mice	Autophagy↑	[69]
*EGCG*						
50 mg/kg	Diabetic cardio-dysfunction	Adipose tissue	MSCs transplantation with oral administration of EGCG	Reduced the cardiac inflammatory markers (p-NFκB, COX2) & fibrosis markers (TGF-β, MMP-9*)*	N/A	[71]
*Resveratrol*						
5 μM	Colitis periodontitis	Periodontal ligament	Injection of RSV-treated MSCs	Inhibited inflammatory T-cell infiltration, enhanced the therapeutic effect of PDLSCs transplantation, recovered epithelial structure in colitis mice	ERK/WNT/FASL	[7]
	Diabetic hepatopathy	Adipose tissue	Injection of RSV-treated MSCs	Reduced apoptosis, suppressed fibrotic pathways, and enhanced the therapeutic effect of ADSCs	SIRT1 /IGF1R↑	[70]
200 mg/kg	Diabetes with podocyte damage	Umbilical cord	MSCs transplantation with oral administration of RSV	Reduced the inflammatory factors MCP-1, RAGE, and NF-κB, protect renal podocyte function, and enhanced the therapeutic effect of hUCMSCs	RAGE-NF-κB ↓	[86]
10 nM	TNF-α Periodontitis	Periodontal ligament	Calcined bovine bone scaffold with RSV-treated PDLSC	Improved osteogenic potential, and facilitated the alveolar bone regeneration in periodontitis rats by PDLSC transplantation therapy	NFκB ↓ SIRT1/Pgc1α ↑ AMPK ↑	[55]
30 mg/kg (i.p injection)	Autoimmune encephalomyelitis	Bone marrow	Injection of RSV-treated MSCs	Enhanced the therapeutic effect of BMSC injection: suppressed pro-inflammatory cytokines (IFN-γ, TNF-α), increased anti-inflammatory cytokines (IL-4, IL-10), promotes a shift in balance from Th1 to Th2 cytokine and reduced T-cell infiltration and in autoimmune encephalomyelitis mice	N/A	[87]

ADSC, adipose-derived mesenchymal stem cells; hUCMSC, human umbilical cord mesenchymal stem cells; MSCs, mesenchymal stem cells; PDLSCs, periodontal ligament-derived mesenchymal stem cells; Th1, T helper type 1; Th2, T helper type 2; EEA, ethanolic extract of astragalus; EGCG, epigallocatechin-3-gallate; OVX, ovariectomy; RSV, resveratrol; AMPK, adenosine monophosphate-activated protein kinase; ERK, extracellular signal-regulated kinases; FASL, FAS ligand; HO-1, heme oxygenase-1; JNK, c-Jun N-terminal protein kinase; NF-κB, nuclear factor kappaB; SIRT 1, sirtuin 1; Pgc1α, peroxisome proliferator-activated receptor gamma coactivator-1α; RAGE, advanced glycation end product receptor

Previous studies have attributed the therapeutic effects of MSCs partially to the ability to migrate into impaired tissues, which regulates a series of cell activities during tissue repair through various signaling pathways. These findings also confirmed that MSCs could secret numerous trophic factors and extracellular vesicles (EVs) in a paracrine or autocrine manner, which is another avenue to enhance the regeneration of the host cells [67, 68]. Therefore, the therapy can be divided into three types, in which NCs can be used to amplify the therapeutic effects of MSC transplantation: (1) to transplant the NCs pre-treated MSCs, (2) to transplant the secreted EVs from NCs pre-treated MSCs, and (3) to transplant MSCs or MSC-derived EVs in accompanying with NCs.

The NCs pre-treated MSCs are able to be transplanted into disease hosts mainly via intravenous injection. The injection of MSCs treated with Osthole restored the immunosuppressive ability of BMSCs, which reduced the lymphocyte infiltration and mucosal damage in experimental inflammatory colitis and prevented cortical bone damage and bone microstructure damage in the osteoporotic mouse model [21]. It also elevated P1NP (procollagen 1 n-terminal peptide), ALP, and reduced TRAP in the serum of OVX (ovariectomy) mice, which had a significant impact on both osteoblastic and osteoclastic activities [69]. The transplantation of RSV-treated ADSCs reduced cell apoptosis and suppressed fibrotic pathways, exhibiting effective tissue repair in diabetic hepatopathy [70]. Parallelly, the transplantation of RSV-treated PDLSCs inhibited inflammatory T-cell infiltration and effectively recovered epithelial structure in colitis mice. In addition, the findings also showed that 10% of the normal amount of PDLSCs pre-treated with RSV could produce a similar effect in colitis treatment, suggesting that NCs enhanced the therapeutic effects and might dramatically reduce the necessary dosage of MSCs for disease treatment [7].

In the radiation-induced intestinal injury rats, the intraperitoneal injection of condition medium of BMSCs pre-treated with Rg1 downregulated the inflammatory cytokines, IL-1β and TNF-α, and promoted the angiogenesis, which was related to a higher release of two pivotal factors VEGF (vascular endothelial growth factor) and IL-6, as well as a critical cytoprotective protein HO-1 (heme oxygenase-1) [68]. These findings indicated that transplantation of the secretome from Rg1 pre-treated MSCs significantly alleviated intestinal damage via the improvement of intestinal regeneration. The ADSC transplantation with oral administration of EGCG showed an obvious reduction in oxidative stress and an effective enhancement in the restoration of cardiac function in diabetic rats [71]. Furthermore, the co-treatment by a sustained-release gel, containing both the EVs from Wharton’s jelly MSCs and the ethanolic extract of astragalus, was found to reduce the expression of inflammatory factors and increase anti-inflammatory cytokine IL-10 in macrophages. Macrophage aggregation was also decreased, as well as the contents of IL-1β and IL-6 in the peripheral blood declined [72]. Based on these findings, MSC transplantation accompanied with the administration of NCs showed a promising therapeutic outcome in disease management; however, the comparison of therapeutic efficacy among these three therapeutic avenues is still lacking in research.

Human oral-derived MSCs (OMSCs) are stem cells isolated from various orofacial tissues, including the periodontal ligament, gingiva, dental pulp, apical papilla, deciduous tooth, dental follicle, and alveolar bone. Compared to other stem cells, OMSCs possess the advantages of abundant sources and easy accessibility. They also hold great potential in tissue regeneration and disease therapies, and have been regarded as superior candidates for various applications and a good cell source for MSC-based cytotherapy [73]. A recent systematic review and network meta-analysis displayed that PDLSCs appeared to be equally effective as BMSCs in stem cell-based therapies for alveolar bone, cementum, and periodontal ligament [74]. These OMSCs also show the multi-potential differentiation capabilities for adipogenic, chondrogenic, myogenic, and neurogenic commitments. Additionally, the ability to regulate immune responses is also similar as other source derived MSCs. These multipotency and immunomodulatory abilities could also be promoted by NC treatment.

It was demonstrated that ginsenoside Rg1 altered 2059 differentially expressed genes in dental pulp stem cells (DPSCs) and influenced cell proliferation and differentiation [75, 76]. The gene ontology (GO) analysis focused on cell proliferation pathways also showed RSV elevated periodontal ligament stem cells (PDLSCs) proliferation through inhibition of cell cycle arrest/apoptosis, as well as the induction of metabolic activity. By analyzing the differentially expressed miRNAs during the osteogenic differentiation, naringin was identified as a common regulator for both DPSC and BMSC under osteo-inductive conditions [77]. Moreover, naringin was reported to facilitate the osteogenesis of PDLSCs both in vitro and in vivo [78], which may be mediated by ERK1/2 signaling [79]. In addition, the extract of Danshen was verified to accelerate the osteogenic differentiation of DPSCs and PDLSCs through ERK1/2 cascades [80, 81]. Wedelolactone was verified to effectively stimulate odontoblast differentiation and mineralization of DPSCs by promoting the Wnt/β-catenin pathway and suppressing NF-κB signaling [49]. The transplantation of Osthole-mediated PDLSC-cell sheets promoted de novo bone formation in the dorsal region of immunocompromised mice than those obtained without Osthole intervention [82], and Osthole upregulated MOZ, MORF, and histone acetylases as key regulators in osteogenic differentiation of PDLSCs [60]. BBR could accelerate odontoblast differentiation of DPSCs by activating the Wnt/β-catenin pathway and promote osteogenesis in PDLSCs via binding to EGFR (epidermal growth factor receptors) on the cell membrane to trigger the intracellular ERK signaling cascades [75, 76]. In a rat in situ regeneration model, RSV enhanced the osteogenic potential of impaired PDLSCs by TNF-α [55, 83]. Taken together, these results confirmed that craniofacial MSCs exhibit a profound ability for tissue regeneration, in which NCs further enhance the therapeutic outcomes via activation of OMSC stemness.

On the other hand, the effect of NCs on the regulation of OMSCs in the immune response is limited. It was found that the EGCG significantly inhibited the inflammatory cytokine expression and apoptosis of DPSCs and PDLSCs under hypoxia injury or LPS inflammatory condition in vitro and exhibited inhibitory effects on pulp tissue inflammation in vivo [84, 85]. In this regard, RSV could dramatically suppress TNF-α induced inflammatory cytokines in PDLSCs and DPSCs, which was partly associated with the inhibitory autophagy-JNK signaling cascades [41]. In the colitis mice, the injection of RSV-treated PDLSCs eliminated the inflammatory cell infiltration and recovered the damaged epithelial layer. All in all, while the evidence implied the abilities of NCs in regulating MSC immunomodulation for disease treatment, further studies are necessary to further explore the underlying mechanisms and optimize MSC-based tissue regeneration and disease therapy.

## Conclusion and perspectives

To summarize, NCs exhibit significant antioxidant and anti-inflammatory effects and are able to regulate the differentiation and immunomodulatory ability of MSCs. While the therapeutic combination of NCs with MSCs is currently still an emerging concept, deeper exploration of the mechanism and further development of technologies might be required for the clinical use of MSCs combined with NCs. In this review, we summarized the current understanding, mainly focusing on the in vitro and animal experiments, of the influence of NCs on MSCs and the related downstream signaling pathways. MSCs combined with NCs could rescue cell viability, restore the function of impaired cells, and promote tissue regeneration in various inflammatory conditions, such as hypoxia, peroxidation, osteoporosis, aging, and periodontitis. The underlying mechanisms are mostly associated with the promotion of MSC stemness through attenuating oxidative or endoplasmic reticulum stress, reducing inflammatory cytokines, suppressing immune cell infiltration, reducing the expression of apoptotic messengers, and supporting contributors to cell proliferation and survival. MSC therapy combined with NCs recently displayed a great therapeutic effect for inflammatory-impaired tissue regeneration, by which OMSCs can be a promising cell resource for the MSC-based cytotreatment. While the underlying mechanism of NCs on the regulation of OMSC immunomodulation is largely left blank, it is an urgent research direction in the field to overcome the Food and Drug Administration (FDA) regulation guidelines for clinical applications. Another challenge that plagues the field of natural compounds is their product purity. As most of the compounds used in the literatures have a purity of 98% or 99% and are commercially available, we believe the conclusions from the literature are still useful to advance the stem cell research field. For future clinical applications, the purity of natural compounds and the specific impurities should be further identified to pass FDA approval.

## Data Availability

Not applicable.
